# A Model for Identifying the Fermentation Degree of Tieguanyin Oolong Tea Based on RGB Image and Hyperspectral Data

**DOI:** 10.3390/foods15020280

**Published:** 2026-01-12

**Authors:** Yuyan Huang, Yongkuai Chen, Chuanhui Li, Tao Wang, Chengxu Zheng, Jian Zhao

**Affiliations:** Institute of Digital Agriculture, Fujian Academy of Agricultural Sciences, Fuzhou 350003, China; 18759871027@163.com (Y.H.);

**Keywords:** Tieguanyin oolong tea, image features, hyperspectral data, feature fusion, fermentation, SHAP interpretability

## Abstract

The fermentation process of oolong tea is a critical step in shaping its quality and flavor profile. In this study, the fermentation degree of Anxi Tieguanyin oolong tea was assessed using image and hyperspectral features. Machine learning algorithms, including Support Vector Machine (SVM), Long Short-Term Memory (LSTM), and Gated Recurrent Unit (GRU), were employed to develop models based on both single-source features and multi-source fused features. First, color and texture features were extracted from RGB images and then processed through Pearson correlation-based feature selection and Principal Component Analysis (PCA) for dimensionality reduction. For the hyperspectral data, preprocessing was conducted using Normalization (Nor) and Standard Normal Variate (SNV), followed by feature selection and dimensionality reduction with Competitive Adaptive Reweighted Sampling (CARS), Successive Projections Algorithm (SPA), and PCA. We then performed mid-level fusion on the two feature sets and selected the most relevant features using L1 regularization for the final modeling stage. Finally, SHapley Additive exPlanations (SHAP) analysis was conducted on the optimal models to reveal key features from both hyperspectral bands and image data. The results indicated that models based on single features achieved test set accuracies of 68.06% to 87.50%, while models based on data fusion achieved 77.78% to 94.44%. Specifically, the Pearson+Nor-SPA+L1+SVM fusion model achieved the highest accuracy of 94.44%. This demonstrates that data feature fusion enables a more comprehensive characterization of the fermentation process, significantly improving model accuracy. SHAP analysis revealed that the hyperspectral bands at 967, 942, 814, 784, 781, 503, 413, and 416 nm, along with the image features H_σ_ and H, played the most crucial roles in distinguishing tea fermentation stages. These findings provide a scientific basis for assessing the fermentation degree of Tieguanyin oolong tea and support the development of intelligent detection systems.

## 1. Introduction

Oolong tea, a representative semi-fermented tea, is known for its distinctive aroma and health benefits, enjoying widespread popularity worldwide [1]. Tieguanyin, a quintessential variety among oolong teas originating from Anxi County, Fujian Province, China, is highly regarded not only for its rich orchid fragrance and unique character but also for its exceptionally intricate processing techniques [2]. The standard production process for Tieguanyin oolong tea involves multiple steps, including withering, fermentation (also referred to as ZuoQing, green-making, or oxidation), pan-firing, shaping, and drying [3]. Among these, fermentation typically comprises 3–4 alternating cycles of tumbling and aeration, which is a critical stage in tea processing [3,4]. Enzymatic oxidation reactions during fermentation directly influence the distribution of polyphenols, theaflavins, and aromatic compounds, thereby altering the color, flavor, and quality of the brewed tea [5,6]. Therefore, insufficient or excessive fermentation can significantly affect the quality of the tea. However, assessing the fermentation degree of Tieguanyin oolong tea currently relies primarily on the experience and subjective judgment of tea masters. This approach is highly subjective and uncertain, making it difficult to meet the demands of modern, standardized, and large-scale production [6,7]. Therefore, there is an urgent need to develop accurate, stable, and intelligent techniques for identifying fermentation degree to advance the digital transformation of the tea processing industry.

In recent years, with technological advancements, non-destructive testing methods such as machine vision and spectroscopy have been applied to tea research. Machine vision technology can acquire visual information from samples, including details about appearance, color, shape, and surface features [8]. Spectral detection technology, as an emerging optical method, contains rich spectral information, enabling the non-destructive detection of internal chemical composition and moisture content with its rapid, objective, and repeatable characteristics [9,10]. By analyzing the absorption patterns of various functional groups (e.g., O-H, C-H, N-H) within molecules, this technology comprehensively reflects sample quality [11]. Research indicates that integrating image and spectral information effectively enhances feature expression, improving model robustness and accuracy [12,13]. Wang et al. classified black tea with varying withering degrees using near-infrared spectroscopy and images, achieving an optimal SVM model classification accuracy of 97.50% [14]. Jin et al. classified four distinct fermentation levels of black tea using spectral-image fusion, achieving an SVR model prediction set accuracy of 89.19% [15]. By integrating near-infrared spectroscopy and computer vision, Ge et al. established a black tea fermentation level discrimination model that achieved 100% accuracy through PCA [16].

While non-destructive techniques like spectroscopy and imaging have been extensively studied for black tea fermentation, research on complex semi-fermented teas like Tieguanyin oolong tea remains relatively limited. On one hand, the oolong fermentation process involves complex dynamic steps such as repeated tumbling and aeration stages, making it difficult to directly transfer fermentation models from other tea types. On the other hand, existing tea fermentation models lack interpretability, hindering the identification of key features contributing to model outcomes and limiting their practical application in production [17,18]. Zheng et al. combined visible and near-infrared (Vis-NIR) spectroscopy and image processing techniques to evaluate three distinct fermentation levels of oolong tea, achieving a 97.14% recognition accuracy with an SVM model [17]. However, their study was restricted to only three fermentation stages, which fails to capture the dynamics of the complete process. Furthermore, the Vis-NIR spectroscopy employed is a point-based technique that captures only localized spectral data, thus lacking spatial comprehensiveness [18].

To overcome these limitations, this study proposes a multi-source data fusion approach for assessing the fermentation degree of Tieguanyin oolong tea. The methodology consisted of the following steps: (1) RGB images and hyperspectral data were collected from tea samples across six distinct fermentation stages, followed by the extraction of color features, texture features, and spectral reflectance data. (2) After applying feature selection and dimensionality reduction, preliminary discriminant models were established using the individual feature sources. (3) A mid-level fusion strategy was implemented, and the final fusion models (SVM, LSTM, GRU) were developed on the feature set optimized by L1 regularization. (4) SHAP explainability analysis was performed on the models to identify the most influential hyperspectral and image features, thereby facilitating the development of intelligent detection systems.

## 2. Materials and Methods

### 2.1. Experimental Design and Sample Selection

Hyperspectral data and RGB images were randomly collected from three different batches during the tea fermentation process on 3–4 May 2024. Samples of the Tieguanyin tea were obtained from the Binghuai Family Farm in Longjuan Township, Anxi County. To investigate over-fermentation, a one-hour extension of the fermentation process was applied following optimal fermentation to establish the over-fermentation stage. The fermentation degree was assessed jointly by three tea masters. Once at least two masters confirmed that the tea had reached a specific fermentation stage, all samples for that stage were collected immediately. The first tumbling lasted 3 min, followed by the first aeration for 50–60 min. The second tumbling lasted 7 min, followed by the second aeration for 3–3.5 h. The third tumbling lasted 15–18 min, followed by the third aeration for 7–7.5 h. A roller-type machine set at 22 revolutions per minute was used for tumbling. For aeration, the leaves were spread 6 cm thick on bamboo trays, which were then placed on racks in a fermentation room maintained at 21 °C.

Data were collected at six key stages of the fermentation process: before the first aeration (Stage 1), at the end of the first aeration (Stage 2), at the end of the second aeration (Stage 3), 3 h after the start of the third aeration (Stage 4), at optimal fermentation (Stage 5), and at over-fermentation (Stage 6). For RGB image data, 400 images were collected per stage. After extracting color and texture features from the images, the feature data from every 10 images were averaged to produce one dataset. Consequently, 40 sets of image feature data were obtained per stage, resulting in a total of 240 sets throughout the fermentation process. Hyperspectral data were sampled 40 times per stage, yielding 240 spectral reflectance datasets in total. The 240 samples, each comprising hyperspectral and image features, were divided into a training set and a test set in a 7:3 ratio using stratified sampling to prevent evaluation bias due to data distribution skewness.

**Figure 1:**
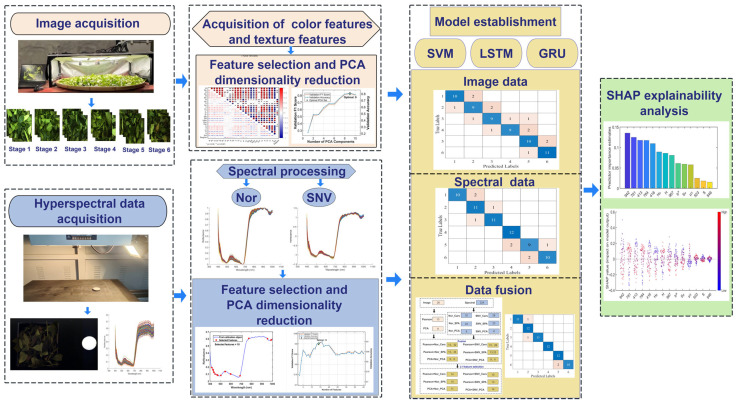
Experimental flowchart.

PCA, Pearson correlation-based feature selection, Competitive Adaptive Reweighted Sampling (CARS), Successive Projections Algorithm (SPA), and L1-based regularization were employed for dimensionality reduction or feature selection. Support Vector Machine (SVM), LSTM (Long Short-Term Memory), and Gated Recurrent Unit (GRU) were used to classify and identify the six fermentation stages of Tieguanyin oolong tea. Prediction models for fermentation degree were developed separately based on single features and on data fusion. The top three models were analyzed using SHAP to identify the most influential features. All model development and performance evaluation were performed using MATLAB R2023b. Figure 1 shows the experimental workflow of this study.

### 2.2. RGB Image Data Acquisition System

**Figure 2:**
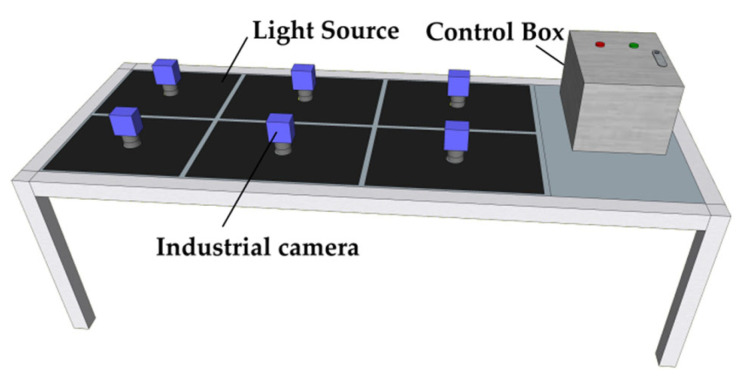
Image data acquisition system.

We independently developed an RGB image data acquisition system (dimensions: 120 cm × 48 cm × 35 cm) that significantly enhances data collection efficiency. The system is equipped with six industrial cameras (T-GE1000C-T-CL, HuaTengVision, Shenzhen, China) and six corresponding 20 cm × 20 cm adjustable flat light sources (JH-FLK200200, Dongguan Juhua Vision Technology Co., Ltd., Dongguan, China). Tea trays can be fed into the system either manually or via a conveyor belt.The maximum resolution of captured images is 3664 × 2748 pixels. Image data acquisition system is illustrated in Figure 2.

### 2.3. Hyperspectral Data Acquisition 

**Figure 3:**
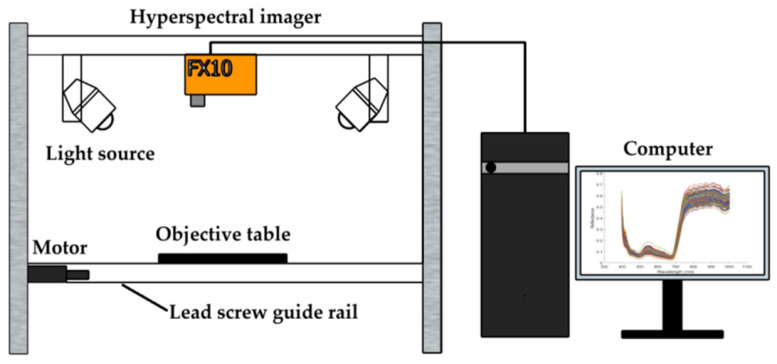
Hyperspectral data acquisition system.

Hyperspectral imaging data of Tieguanyin tea samples were acquired using a visible-near-infrared hyperspectral imaging system (FX10, SPECIM, Oulu, Finland). The system comprises a hyperspectral imager, light source, motor, lead screw guide rail, objective table, and computer (as shown in Figure 3). The light source uses four 150 W halogen lamps (Wenzhou Aolijia Electric Appliance Co., Ltd., Wenzhou, China). The hyperspectral camera covers a spectral range of 400–1000 nm with 224 spectral bands.

Prior to data acquisition, the hyperspectral imaging system was warmed up for 30 min to ensure stable operation. The motorized objective table operates at a speed of 2 mm/s. The vertical distance between the hyperspectral camera and the sample is maintained at 40 cm. For each scan, a 60 g tea sample was evenly distributed in a black square container (14 cm × 14 cm × 5 cm, L × W × H).

### 2.4. Data Acquisition

#### 2.4.1. RGB Image Feature Extraction

Image feature extraction involved the extraction of color features and texture features. For color features, 15 parameters were extracted from three color spaces (RGB, HSI, and Lab) of each image, including mean red value (R), mean green value (G), mean blue value (B), red standard deviation (R_σ_), green standard deviation (G_σ_), blue standard deviation (B_σ_), mean hue (H), mean saturation (S), mean intensity (I), hue standard deviation (H_σ_), saturation standard deviation (S_σ_), intensity standard deviation (I_σ_), mean lightness component (L*), mean red-green component (a*), and mean blue-yellow component (b*) [19]. For texture features, first, RGB images were converted to grayscale images, and 6 texture feature parameters based on grayscale statistical moments were extracted, including mean gray level (m), standard deviation (σ), smoothness (Sm), third moment (µ_3_), uniformity (U), and entropy (**e**) [20,21,22]. Additionally, a Gray-Level Co-occurrence Matrix (GLCM) was calculated based on the grayscale images to obtain five additional texture feature parameters: energy (EN), dissimilarity (Dis), contrast (Con), correlation (Cor), and homogeneity (Hom) [23,24,25]. All image processing was implemented in Python 3.8, yielding a total of 26 color and texture features.

#### 2.4.2. Hyperspectral Data Acquisition and Preprocessing Algorithm

The mean spectrum from each Region of Interest (ROI) was extracted using ENVI 3.5 software. A rectangular ROI was selected from the center of each tea sample, and the average value within this region was used as the spectral value for that sample. This approach ensured that the extracted data represented the entire sample while avoiding the edges of the container, thereby effectively capturing the comprehensive spectral information of the tea leaves [26]. To mitigate the impact of uneven light intensity and dark current on the acquired information, the hyperspectral images were calibrated using the following formula [27]:

$$R\;=\;\frac{R_{\mathrm{raw}\;}-\;B}{W\;-\;B}$$

In this formula, R denotes the calibrated hyperspectral image. R_raw_ represents the raw hyperspectral image, while W and B denote the white reference image and dark current image, respectively.

To effectively suppress adverse effects from baseline drift, noise, and stray light, two classical preprocessing algorithms were applied to the raw spectral data: Standard Normal Variate (SNV) transformation was used to eliminate scattering effects between samples, and Normalization (Nor) was employed to standardize the data scale [14].

### 2.5. Dimension Reduction and Feature Extraction Algorithms

In this study, the image data contains 26 feature variables, while the hyperspectral data includes reflectance information across 224 bands. Although there is sufficient information to evaluate the fermentation degree, these variables may exhibit high redundancy. This will affect the computational speed and accuracy of the model, and increase the risk of overfitting [28,29]. Therefore, selecting effective variables to assess the fermentation degree is particularly important.

PCA is a commonly used method for data dimensionality reduction [30]. It projects the original data matrix onto selected feature vectors to obtain the most valuable principal components (PCs), which can represent the majority of the sample information. The number of PCs was selected according to the following criteria. First, PCs with a cumulative contribution rate exceeding 99% were retained. Then, 5-fold cross-validation (CV) was performed on the training set using an SVM model (C = 1) to evaluate models built with different numbers of PCs from the first step. The optimal number of PCs was determined based on the following priorities: the highest average accuracy from the 5-fold CV was given primary importance, followed by a higher average F1-score, and finally, the smallest number of PCs when performance was comparable.

Feature selection serves as another strategy to eliminate irrelevant variables, enhancing both model speed and accuracy [31]. It directly selects the optimal subset from the original feature set while preserving the original semantic meaning of features, thereby improving model interpretability and reliability [32]. This paper employs Pearson correlation analysis for image feature selection and CARS and SPA for spectral feature selection. Following feature fusion, an L1-regularization-based feature selection was further applied. The strategy was implemented as follows: First, Lasso regression (α = 1) was applied to the training set to generate a series of candidate feature subsets with varying levels of sparsity (i.e., different numbers of features). Subsequently, for each candidate subset, a linear SVM (C = 1) was evaluated using 5-fold CV on the training set, and the average validation accuracy along with the macro-average F1-score were computed. The criterion for selecting the optimal feature subset followed the same principle established in the PCA stage: prioritizing the highest average CV accuracy, then the higher macro F1-score among models with comparable accuracy, and finally selecting the model with the fewest features to ensure parsimony.

The configurations for the other feature selection methods were as follows:

Pearson Correlation Analysis: The correlation coefficients between all 26 image features and the fermentation stage were calculated. Features with an absolute correlation coefficient greater than 0.1 were retained.

CARS: This algorithm selects key spectral features through iterative selective sampling and weighting of regression coefficients, effectively reducing dimensionality while preserving critical information. It was configured with 100 iterations and a Monte Carlo sampling ratio of 80%. Partial Least Squares regression was employed, treating the ordinal class labels (1–6) as a continuous response variable—a common practice in spectroscopy for handling ordinal classification problems [33]. The subset yielding the minimum Root Mean Square Error of Cross-Validation was selected.

SPA: SPA selects feature wavelengths sequentially through vector projection analysis, aiming to minimize collinearity by iteratively choosing variables with the least redundant information. In this study, the minimum and maximum numbers of variables to select were set as follows: the minimum (N_min) was fixed at 10, while the maximum (N_max) was set to the lesser of (N − 1) (where N is the sample size) and the total number of available spectral variables. The algorithm automatically determined the final number of variables by minimizing the root mean square error of cross-validation.

### 2.6. Data Fusion

**Figure 4:**
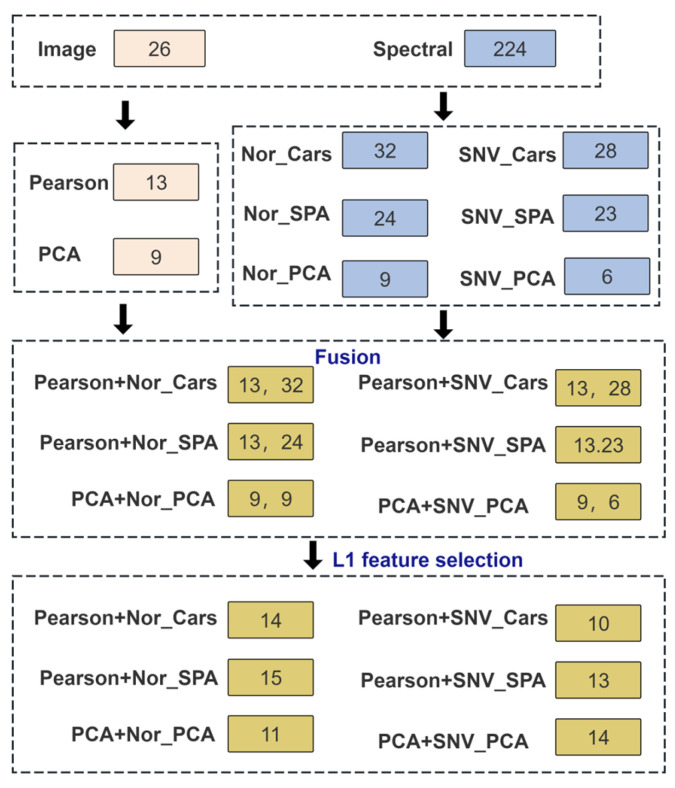
The flow diagram of data fusion strategy.

Data fusion is a strategy that integrates valuable information from diverse sources to enhance the comprehensiveness of information utilization [34]. Data fusion strategies can be categorized into three levels: low, medium, and high. In low-level fusion, all information from different data sources is simply concatenated into a new matrix. Mid-level fusion extracts key feature data from each individual information source to form a new matrix for building predictive models [28]. Compared to low- and mid-level fusion, high-level fusion is less common and demands a high degree of independence of each information source. Crucially, information can be easily lost if the correlation between data across information sources is ignored [14]. Therefore, this study adopts a mid-level fusion strategy to evaluate the fermentation degree of oolong tea. After extracting key features from each individual information source, a new matrix is formed. Subsequently, L1 feature selection is applied to further select important features for modeling. The overall data fusion strategy is illustrated in Figure 4.

### 2.7. SHAP Explainability Analysis

SHAP (SHapley Additive exPlanations) employs the Shapley value method, a fair allocation algorithm from game theory, to quantify each feature’s contribution to model predictions [35]. SHAP values account for interactions between features, providing a comprehensive perspective on feature importance. We calculated the SHAP values using the built-in Shapley function in MATLAB, which is implemented based on the KernelSHAP algorithm. The background dataset was set as the training set (comprising 168 samples in total). During the KernelSHAP computation, the default sampling number was applied, i.e., 2 × M + 2048 times, where M represents the number of features. SHAP values were computed for all 72 samples in the test set, and the features were ranked by their mean absolute SHAP values to identify the key features. SHAP analysis identifies key features from spectral bands and image data, providing theoretical support for the intelligent detection of tea fermentation degree.

### 2.8. Models and Evaluation Metrics

#### 2.8.1. Models

This study employed three common models for analysis: SVM, LSTM, and GRU. SVM is a supervised learning method based on maximum margin theory, exhibiting strong generalization capabilities with low dependence on sample size [36,37]. SVM exhibit robust performance and are less prone to overfitting when handling small-sample, high-dimensional data, such as spectral features. LSTM is a specialized recurrent neural network architecture. By incorporating gating mechanisms, it effectively alleviates the vanishing gradient problem common in traditional RNNs when modeling long sequences. This enables accurate prediction of optimal fermentation timing and identification of critical stages such as under-fermentation or over-fermentation [38]. GRU is a lightweight variant of LSTM. By merging the forget gate and input gate into an update gate and simplifying the memory cell structure, it significantly reduces computational complexity while maintaining temporal dependency modeling capabilities [39]. The tea fermentation process is a temporal sequence of physicochemical changes, making its stage classification a quintessential sequential problem. Temporal models like LSTM and GRU are particularly suited for this task due to their inherent ability to capture long-range dynamic dependencies, which are crucial for distinguishing between evolving process states.

#### 2.8.2. Model Hyperparameter Tuning and Validation Strategy

For the SVM model, an RBF kernel was used. Its hyperparameters, the penalty parameter C and the kernel coefficient γ, were optimized via grid search with 5-fold cross-validation on the training set. The search grids were C ∈ {16, 32, 64, 128, 256, 512} and γ ∈ {0.001, 0.002, 0.004, 0.008, 0.016, 0.032, 0.064, 0.128}. The combination maximizing the average CV accuracy was selected. Finally, the model was retrained on the entire training set with these optimal parameters.

Both the LSTM and GRU models were implemented with a two-layer architecture, each comprising 16 hidden units. They were trained using the Adam optimizer (learning rate: 0.001, batch size: 8). To prevent overfitting, a Dropout layer (rate = 0.3) was applied, and the ReLU activation function was used. The optimal number of training epochs was determined through 5-fold CV on the training set, where the epoch yielding the highest validation accuracy within each fold was identified as the early stopping point. Subsequently, the model was retrained on the entire training set for this optimal epoch count, and its final generalization performance was assessed on the independent test set.

#### 2.8.3. Model Evaluation Metrics

The performance of the Tieguanyin fermentation stage classification model was comprehensively evaluated using a confusion matrix and core classification metrics derived from it, specifically including: Accuracy (Acc), Precision (Pre), Recall (Rec), F1-score, and Specificity. To further assess the statistical reliability of the model accuracy, uncertainty analysis was performed using the Bootstrap resampling method (with 1000 repetitions) to estimate the 95% confidence interval (CI) for the accuracy metric.

## 3. Experimental Results and Analysis

### 3.1. Fermentation Degree Model Based on Image Features

#### 3.1.1. Image Feature Selection

**Figure 5:**
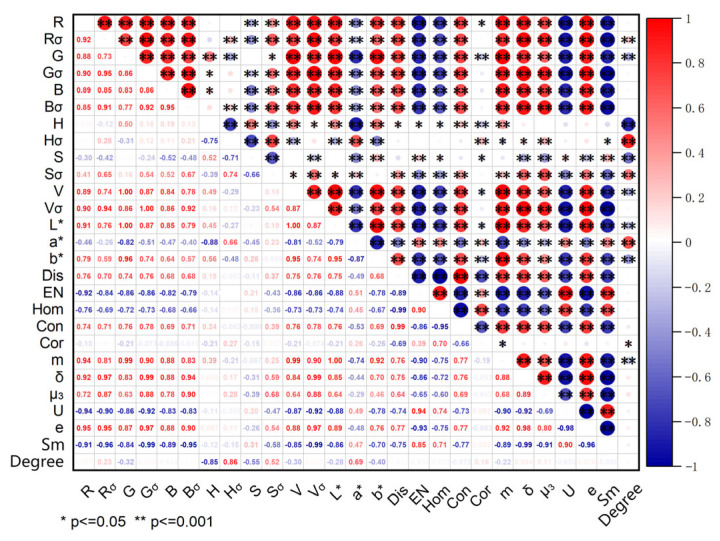
Pearson correlation analysis result.

Pearson correlation analysis was conducted between 26 image feature values and fermentation degree, with results shown in Figure 5. Darker circle colors indicate higher correlation coefficients (r), where red denotes positive correlation and blue denotes negative correlation. The Pearson correlation analysis indicates a significant association between image features and fermentation degree. H_σ_ exhibits a strong positive correlation with fermentation degree (r = 0.86), while H shows a strong negative correlation (r = −0.85). This aligns closely with the formation process of Anxi Tieguanyin’s characteristic “green leaves with red edges.” During fermentation, leaf edge friction and damage caused by tumbling bring polyphenol oxidase (PPO) in cells into contact with tea polyphenols. This promotes the oxidation and polymerization of tea polyphenols, generating colored substances such as theaflavins and thearubigins. Consequently, leaf edges turn red while the leaf centers remain green, and the increased color contrast leads to a rise in H_σ_. As fermentation progresses, the overall dominant hue of the leaves shifts from emerald green to deep green and reddish-brown, corresponding to a decrease in H [18]. Fermentation degree shows a positive correlation with a* (r = 0.69), reflecting the shift toward magenta as leaves oxidize. It correlates negatively with S (r = −0.55) and positively with S_σ_ (r = 0.52). During the fermentation process, the oxidation levels vary significantly across different parts of the leaf, leading to a notable increase in the overall difference in saturation. As a result, S_σ_ exhibits a positive correlation with fermentation degree—meaning the higher the fermentation degree, the greater the saturation fluctuation. In the later stages of fermentation, with the deepening of overall oxidation, tea leaves undergo browning, and the color gradually darkens, resulting in a decrease in overall saturation. Therefore, S is negatively correlated with the degree of fermentation.

Based on the correlation analysis results between image features and fermentation degree, the overall trends and change mechanisms align with the actual color and physicochemical transformations during the fermentation process of Anxi Tieguanyin. This validates the feasibility of quantifying the fermentation degree through image features, while also providing theoretical support for further optimizing non-destructive testing technologies. Following correlation-based feature selection, the following 13 image features with correlation coefficients exceeding 0.1 were retained for modeling: H_σ_, H, a, S, S_σ_, b, G, V, L*, R_σ_, m, Cor, and µ_3_. Their correlation coefficients are 0.86, −0.85, 0.69, −0.55, 0.52, −0.40, −0.32, −0.30, −0.28, 0.23, −0.22, 0.16, and 0.11, respectively.

#### 3.1.2. PCA Dimension Reduction in Image Features

**Figure 6:**
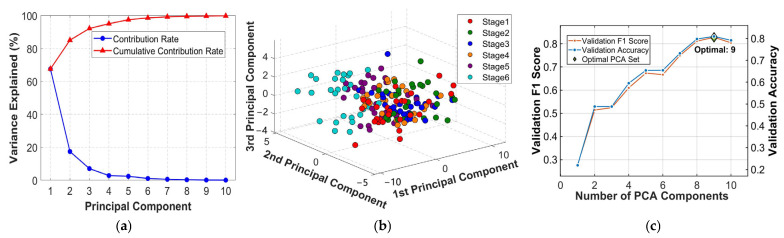
PCA analysis results of image features: (**a**) PCA contribution rate plot; (**b**) the three-dimensional scatter plot of the first three principal components; (**c**) number of PCs and SVM model performance.

PCA was applied to the image features. The PCA contribution rate plot is shown in Figure 6a, where the blue curve represents the contribution rate of a single principal component, and the red curve reflects the cumulative contribution rate. The cumulative contribution of the first 10 principal components exceeds 99%, with PC1 contributing 67.63%, PC2 17.47%, and PC3 7.10%. The cumulative contribution of the first three principal components reaches 92.20%. The three-dimensional scatter plot of the first three principal components is shown in Figure 6b. Although there exists a certain overlapping region among samples with different fermentation degrees, they already exhibit a certain level of distinguishability. Stage 1 (pre-fermentation) exhibits relatively concentrated red points, indicating a certain degree of clustering in image features within the relative space. This suggests that differences in image features are minimal prior to fermentation. As fermentation progresses, points gradually disperse into distinct regions, reflecting continuous changes in image features during fermentation and increasing stage-specific differentiation. For instance, Stage 5 purple points are relatively clustered, indicating stable features in moderately fermented images. Stage 6 points exhibit relatively independent distribution with noticeable separation from other stages, demonstrating effective identification of over-fermented images. Based on the training set, 5-fold CV was performed using an SVM model to determine the optimal number of principal components. The results indicated that using nine PCs yielded the highest average accuracy and F1-score on the validation set. As shown in Figure 6c, an appropriate number of principal components can enhance the discrimination performance for tea fermentation stages. Consequently, nine PCs were selected for all subsequent modeling.

#### 3.1.3. Model Results Based on Image Features

To discriminate the fermentation degree of oolong tea, three machine learning algorithms—SVM, LSTM, and GRU—were applied to construct classification models. These models were built using raw image features, features selected via Pearson correlation, and features reduced via PCA, respectively. The performance metrics of all models are shown in Table 1.

**Figure 7:**
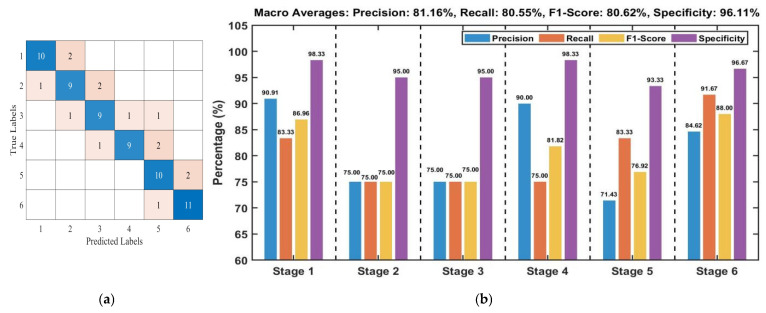
Test set performance evaluation of the Pearson+SVM model based on image features: (**a**) Confusion matrix; (**b**) Per-stage model evaluation metrics.

Test set results indicate that the classification accuracies achieved by the three algorithms are generally comparable, ranging from 76.39% to 80.56%. The Pearson+SVM, Pearson+LSTM, Pearson+GRU, and PCA+LSTM models achieved the highest test set accuracy of 80.56%. Figure 7a presents the confusion matrix for the Pearson+SVM model, while Figure 7b details the performance metrics for each stage. The confusion matrix reveals that misclassifications primarily occur between adjacent fermentation stages, which is likely attributable to the high similarity in image features among neighboring stages. An analysis of the per-stage metrics in Figure 7b indicates varied classification efficacy. Stages 1 and 6 exhibited the strongest performance: Stage 1 achieved a Precision of 90.91% and an F1-Score of 86.96%, while Stage 6 attained a Recall of 91.67% and an F1-Score of 88.00%. Moreover, the Specificity for both stages exceeded 96%, demonstrating the model’s robust ability to correctly reject samples from non-target stages.

As demonstrated by the results in Table 1, performing feature selection or dimensionality reduction on image data before model training is an effective preprocessing strategy. It reduces dimensionality, enhances computational efficiency, and maintains strong model discriminative ability, thereby fulfilling the goals of feature extraction and reduction. In subsequent work, the feature-selected and dimensionally reduced image data will also be used for data fusion modeling.

### 3.2. Fermentation Degree Model Based on Hyperspectral Features

#### 3.2.1. Hyperspectral Data Preprocessing Analysis

**Figure 8:**
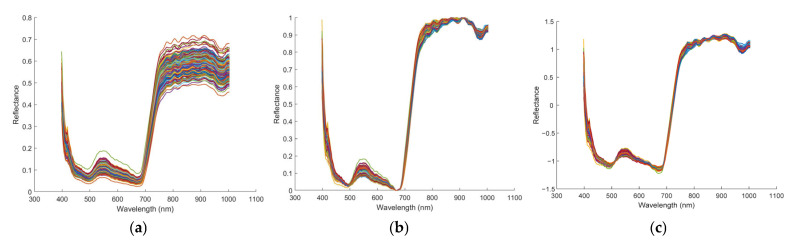
The raw spectra and preprocessed spectra: (**a**) raw; (**b**) Nor preprocessings; (**c**) SNV preprocessing.

To mitigate spectral fluctuations and eliminate irrelevant scattering effects, Nor and SNV transformations were applied to the raw spectra. The raw and preprocessed spectral curves are presented in Figure 8. As shown in Figure 8b,c, both Nor and SNV preprocessing effectively reduced inter-sample variation and resulted in smoother spectral curves.

#### 3.2.2. Extraction of Hyperspectral Feature Bands

**Figure 9:**
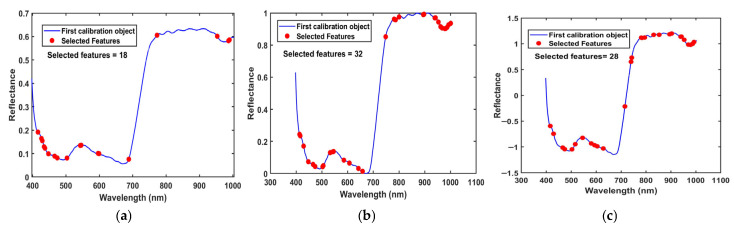

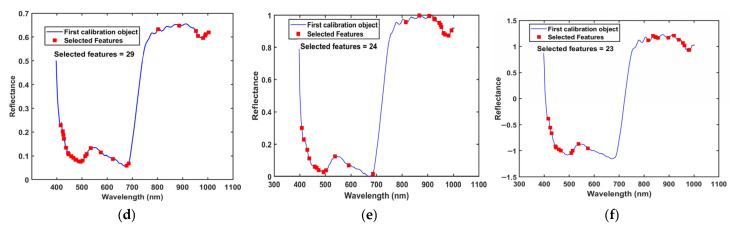
Feature bands selected by CARS and SPA algorithms: (**a**) raw-CARS; (**b**) Nor-CARS; (**c**) SNV-CARS; (**d**) raw-SPA; (**e**) Nor-SPA; (**f**) SNV-SPA.

To eliminate interference from irrelevant bands on model accuracy, both CARS and SPA feature wavelength screening algorithms were employed for automatic feature extraction from the hyperspectral data, with the results shown in Figure 9. The number of selected feature bands ranged from 18 to 32. Among these, the spectral data retained only 18 wavelengths after CARS screening, accounting for 8.03% of the total wavelengths. The maximum number of bands (32, accounting for 14.29%) was obtained after Nor preprocessing followed by CARS filtering. The screening results indicate that the feature bands are primarily concentrated in the 400–500 nm and 750–1000 nm regions, demonstrating that these two spectral intervals exhibit strong sensitivity and discrimination capabilities for the tea fermentation process.

#### 3.2.3. PCA Dimensionality Reduction in Hyperspectral Data

**Figure 10:**
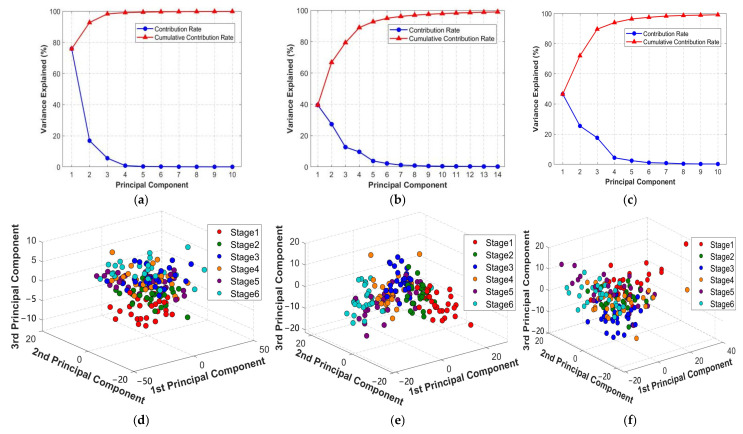

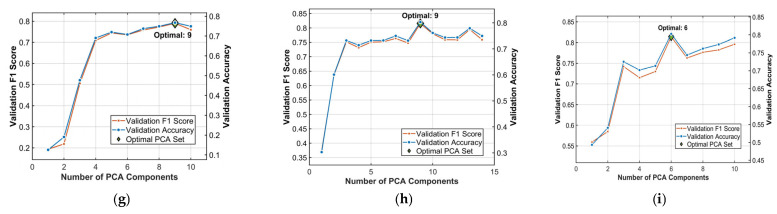
PCA contribution rate plots of spectral features: (**a**) raw; (**b**) after Nor preprocessing; (**c**) after SNV preprocessing. 3D scatter plots of the first three principal components: (**d**) raw; (**e**) after Nor preprocessing; (**f**) after SNV preprocessing. Number of PCs and SVM model performance: (**g**) raw; (**h**) after Nor preprocessing; (**i**) after SNV preprocessing.

PCA was applied for dimensionality reduction to the raw spectral data as well as the data preprocessed by Nor and SNV. The results of the dimensionality reduction are shown in Figure 10a–c. The first several principal components with a cumulative contribution rate exceeding 99% were obtained. Specifically, the cumulative contribution rate reached 99% with the first 10, 14, and 10 principal components for the raw, Nor, and SNV data, respectively.

The three-dimensional scatter plots of the first three principal components are shown in Figure 10d–f. Compared to the raw data, the Nor and SNV preprocessed data exhibited a marginal improvement in clustering, with more distinct separation between different fermentation stages. The relationship between the number of principal components selected and the performance of the SVM model is shown in Figure 10g–i. The SVM model achieved its optimal performance on the test set when 9, 9, and 6 principal components were selected from the raw, Nor, and SNV data, respectively. Consequently, these numbers of PCs will be used for subsequent modeling.

#### 3.2.4. Hyperspectral Data Modeling

**Figure 11:**
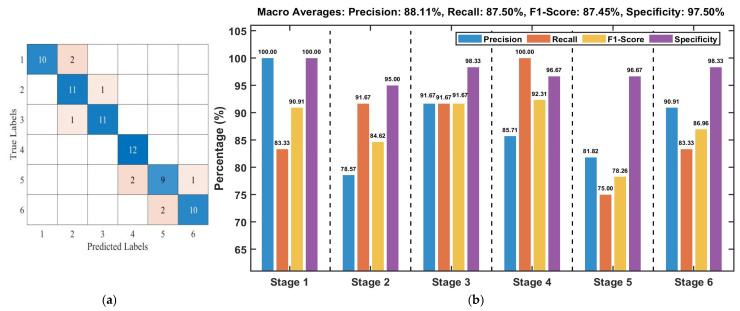
Evaluation metrics of the Nor-SPA+SVM model based on spectral features on the test set: (**a**) Confusion matrix; (**b**) Per-stage model evaluation metric.

Fermentation degree models were developed using SVM, LSTM, and GRU algorithms, based on raw, Nor, and SNV spectral data in combination with CARS, SPA feature selection, and PCA dimensionality reduction. The performance of these models is summarized in Table 2. The results indicate that the test set accuracy across all models ranged from 68.06% to 87.50%. The Nor-SPA+SVM model achieved the highest accuracy of 87.50%. The confusion matrix is presented in Figure 11a, with detailed per-class metrics provided in Figure 11b. Analysis of Figure 11a reveals that all misclassifications are confined to adjacent stages, with no instances of severe error spanning two or more stages. As shown in Figure 11b, classification performance varies across stages: stage 3 exhibits the most balanced and excellent performance, with Precision, Recall, and F1-Score all reaching 91.67%, and a high Specificity of 98.33%. Stage 4 achieves perfect Recall and Specificity, indicating flawless identification of all target samples and accurate rejection of non-target samples. Its Precision (85.71%), however, shows a slight decrease, which is consistent with the observed inter-stage confusion. Stage 5 demonstrates relatively weaker performance, registering the lowest Recall (75%) and F1-Score (78.26%) among all stages. Stages 1, 2, and 6 all maintain strong performance across metrics, with each achieving an F1-Score above 84%. Notably, the Specificity for every stage remains at 95% or higher, indicating the model’s consistently robust capability to distinguish samples from non-target stages overall.

Except for the SNV-PCA+SVM and SNV-PCA+GRU models, Nor and SNV preprocessing delivered superior or equal test set accuracy compared to raw spectral data for all models built. Therefore, Nor and SNV preprocessing demonstrate a clear advantage over using raw spectral data. Consequently, the preprocessed data from these methods will be selected for subsequent integrated data modeling.

### 3.3. Fermentation Degree Model Based on Fusion Data

**Figure 12:**
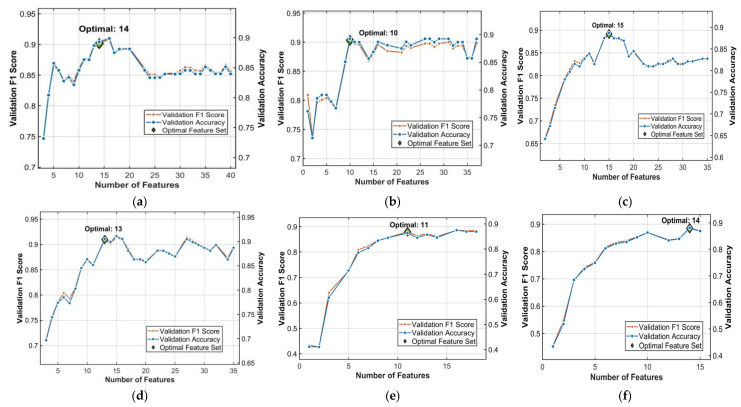
Number of L1-selected features and model performance. (**a**) Pearson+Nor-CARS+L1+SVM; (**b**) Pearson+SNV-CARS+L1+SVM; (**c**) Pearson+Nor-SPA+L1+SVM; (**d**) Pearson+SNV-SPA+L1+SVM; (**e**) PCA+Nor-PCA+L1+SVM; (**f**) PCA+SNV-PCA+L1+SVM.

Hyperspectral data captures subtle changes in the microstructure and chemical composition of tea leaves during fermentation, while image features reflect the associated color and texture alterations [20]. Integrating these data types provides a richer feature set for model training. The image data was processed by Pearson feature selection and PCA dimensionality reduction, while the spectral data was preprocessed with Nor and SNV, followed by feature selection (CARS and SPA) and PCA. Following the fusion of image and spectral data, L1 regularization was applied for feature selection to further reduce redundancy. The number of retained features was determined based on the performance of the SVM model on the validation set. Figure 12 illustrates the number of L1-selected features and the corresponding performance for all six models. Taking the Pearson+Nor-CARS+L1+SVM model as an example, L1 selection reduced the feature set from 45 to 14, which concurrently improved both accuracy and F1-score on the validation set. Therefore, L1 feature selection effectively achieved the objectives of reducing redundancy, enhancing accuracy, and mitigating overfitting.

**Figure 13:**
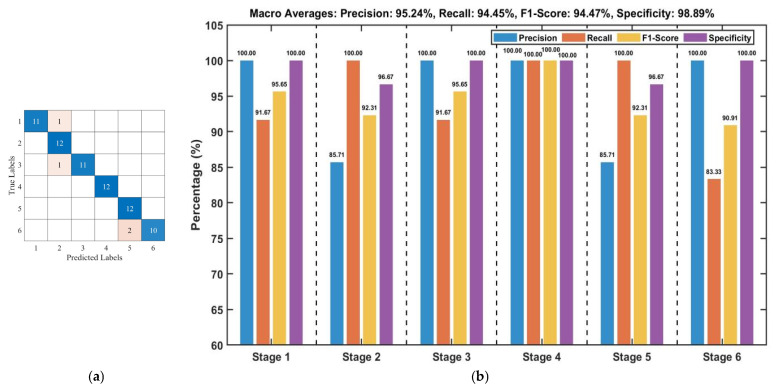
Evaluation metrics of the Pearson+Nor-SPA+L1+SVM Model based on fusion features on the Test Set: (**a**) Confusion matrix; (**b**) Per-stage model evaluation metric.

Following data fusion and L1 feature selection, classification models based on SVM, LSTM, and GRU were constructed. As summarized in Table 3, the test accuracy of all models ranged from 77.78% to 94.44%. Overall, the traditional machine learning model (SVM) outperformed the deep learning models (LSTM and GRU) in classification performance. The best-performing model, Pearson+Nor-SPA+L1+SVM, achieved a test accuracy of 94.44%. Its confusion matrix is shown in Figure 13a, and the performance metrics for each stage are detailed in Figure 13b. Analysis of Figure 13a indicates that the model produces very few misclassifications, and these are entirely confined to adjacent stages. Figure 13b reveals that Stage 4 attained perfect scores (100%) for Precision, Recall, and F1-Score. While minor misclassifications in a few other stages led to slight fluctuations in either Recall or Precision, their F1-Scores remained consistently high, above 90%. In conclusion, the model demonstrates strong discriminative capability for most fermentation stages, with confusion occurring only minimally between a few adjacent stages that share highly similar features.

**Figure 14:**
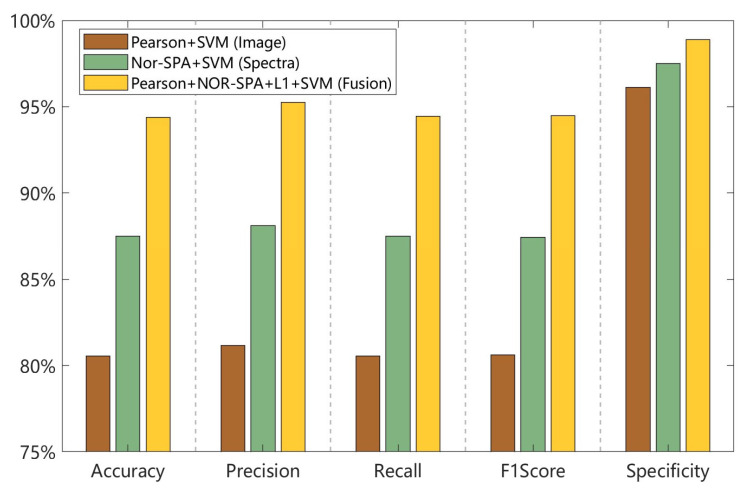
Performance of different models on the test set.

To evaluate the effectiveness of feature fusion, we compared the top-performing models based on single features and fused features on the test set. The results, presented in Figure 14, show that the models relying on a single data source performed the worst across all evaluation metrics. In contrast, the fusion model significantly outperformed both the image-based and spectral-based models in accuracy, precision, recall, F1-score, and specificity. This demonstrates that multimodal data fusion substantially enhances classification robustness. Furthermore, by integrating image and spectral information with appropriate feature processing and machine learning models (particularly SVM), our approach achieved high-accuracy identification of the six fermentation stages. This work thus provides a practical and effective methodology for the intelligent assessment of the fermentation degree during tea processing.

### 3.4. SHAP Interpretability Analysis

To further investigate the contribution of fused spectral and image features to the prediction of oolong tea fermentation stages, SHAP analysis was performed on the three best-performing models (Pearson+Nor-CARS+L1+SVM, Pearson+SNV-CARS+L1+SVM, and Pearson+Nor-SPA+L1+SVM), which achieved test accuracies ranging from 93.06% to 94.44%. This analysis was conducted to interpret the importance of individual features within the models.

**Figure 15:**
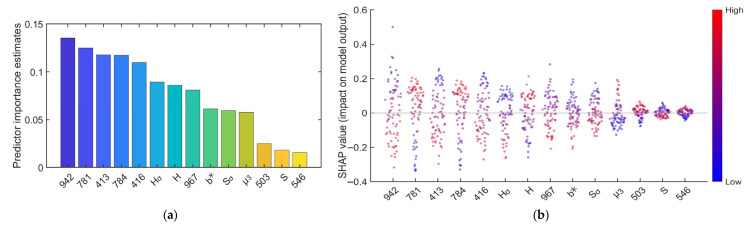

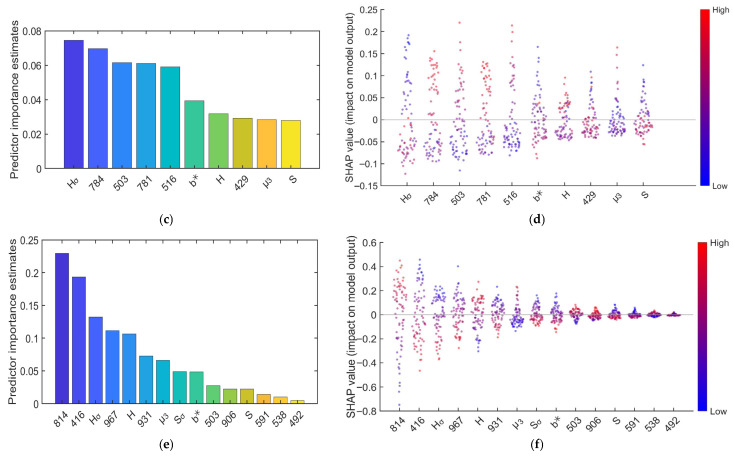
SHAP analysis results of the models: (**a**) Feature importance bar plot of the Pearson+Nor-CARS+L1+SVM model; (**b**) SHAP scatter plot of the Pearson+Nor-CARS+L1+SVM model; (**c**) Feature importance plot of the Pearson+SNV-CARS+L1+SVM model; (**d**) SHAP scatter plot of the Pearson+SNV-CARS+L1+SVM model; (**e**) Feature importance plot of the Pearson+Nor-SPA+L1+SVM model; (**f**) SHAP scatter plot of the Pearson+Nor-SPA+L1+SVM model.

Figure 15a,b present the feature importance ranking and SHAP scatter plot for the Pearson+Nor-CARS+L1+SVM model. The bar chart shows that among the 14 final fused features, the most influential features are 942 nm, 781 nm, 413 nm, 784 nm, 416 nm, H_σ_, and H. The SHAP scatter plot further reveals that most high-contribution features display a clear “red-blue separation”. This pattern indicates that feature values have a definitive directional impact (positive or negative) on the predictions. For example, for the 781 nm band, high feature values (red) are associated with positive SHAP values, suggesting that higher reflectance at this wavelength leads the model to predict a higher fermentation degree. Conversely, the image feature H_σ_ shows a strong negative correlation, where higher H_σ_ values correspond to predictions of a lower fermentation stage. The feature importance and SHAP plots for the Pearson+SNV-CARS+L1+SVM model are shown in Figure 15c,d, which identify H_σ_, 784 nm, 503 nm, and 781 nm as the most critical features. These features play a dominant role in the model’s discrimination of fermentation stages. Similarly, the SHAP plot for this model also shows a distinct “red-blue separation” for its top features. Figure 15e,f display the results for the Pearson+Nor-SPA+L1+SVM model. The importance ranking highlights 814 nm, 416 nm, H_σ_, 967 nm, and H as the top five most influential features, with contributions significantly surpassing the others. These are the key factors enabling accurate fermentation stage classification. Again, a pronounced “red-blue separation” is observed for these primary features.

In summary, the SHAP analysis across these three SVM models reveals that specific spectral bands—including long-wave bands at 781, 784, 814, 942, and 967 nm, and short-wave bands at 413, 416, and 503 nm—play a crucial role in distinguishing tea fermentation stages. At the same time, image features such as H and Hσ also played a crucial role in the discrimination of the tea fermentation process. The specific mechanisms are as follows:

Negative contribution of long NIR bands (942 nm and 967 nm): This is primarily due to moisture loss and state changes in leaves during fermentation. These bands lie in the sensitive region of the third overtone of O–H stretching vibrations, making them highly responsive to variations in water content and hydrogen-bond networks [40]. The migration and reduction in moisture directly enhance light absorption in this region, leading to decreased reflectance.

Positive contribution of long NIR bands (781, 784, and 814 nm): This is mainly attributed to increased light scattering caused by the physical withering of leaf structure during fermentation [41]. Tumbling and water loss lead to cell-structure collapse and tissue loosening, significantly raising the probability and path length of light scattering inside the leaves, thereby increasing reflectance.

The blue-violet bands (413 and 416 nm) showed significant negative SHAP contributions, indicating decreased reflectance with advancing fermentation. This results from the holistic transformation of leaf optical properties during fermentation: while chlorophyll degradation reduces its characteristic absorption around 430 nm, this effect is outweighed by the broad-spectrum absorption of newly formed dark pigments (e.g., theaflavins, thearubigins) and the enhanced light absorption due to tissue breakdown. Thus, the reflectance decline directly marks the biochemical and physical synergistic darkening of the leaf.

Regarding image-based features, the SHAP results align with the Pearson correlation analysis of image characteristics, both highlighting the typical “green leaf with red edges” morphology of Tieguanyin tea. As fermentation proceeds, the leaf edges turn reddish due to localized deep oxidation, while the center retains its green color. This increases the dispersion of hue distribution (higher Hσ) and darkens the overall color (lower H), demonstrating that the model effectively captures the “red-edge green-core” pattern—a visual signature of spatial heterogeneity in fermentation—and uses Hσ and H as key indicators for determining the fermentation stage.

This consolidated finding indicates that these specific spectral and color features are the core elements driving the performance of the fusion model. It provides valuable insights for future model refinement and a deeper understanding of the discriminative mechanism underlying tea fermentation.

## 4. Conclusions

This study presents an intelligent model for discriminating Tieguanyin tea fermentation stages through the fusion of images and hyperspectral data. The proposed approach exhibits superior capabilities in real-time data collection, multi-level feature fusion, and model interpretability compared to existing methods.

A dedicated six-channel image acquisition system was constructed to rapidly capture tea leaf images, from which color and texture features were extracted. Models based solely on image features achieved test accuracies ranging from 76.39% to 80.56%, with the Pearson+SVM, Pearson+LSTM, Pearson+GRU, and PCA+LSTM models all reaching 80.56%.

Unlike traditional point-based Vis-NIR spectroscopy, this study utilizes a hyperspectral imaging system spanning 400–1000 nm across 224 spectral bands. This approach captures complementary spatial and spectral information at high resolution, thereby significantly enhancing the comprehensiveness and robustness of feature representation. To reduce noise, the raw spectral data was preprocessed with Nor and SNV, followed by feature selection (CARS and SPA) and PCA. Models based on spectral data achieved test set accuracies of 68.06% to 87.50%, with the Nor-SPA+SVM model achieving the highest accuracy of 87.50%. These results indicate that while single-modality models can effectively discriminate fermentation stages, there remains considerable potential for improving their accuracy.

By fusing image and spectral features, model performance was significantly enhanced, with test set accuracy reaching 77.78% to 94.44%. Notably, the Pearson+Nor-SPA+L1+SVM model achieved a peak accuracy of 94.44%. This demonstrates that data fusion provides a more comprehensive characterization of the fermentation process, thereby significantly improving the model’s discriminative power.

At the model interpretation level, this study introduced SHAP explainability analysis for the first time in oolong tea fermentation research, clearly revealing the key spectral bands and image features that influence classification decisions, thereby enhancing model transparency and credibility. SHAP analysis demonstrated that specific hyperspectral bands such as 967, 942, 814, 784, 781, 503, 413, and 416 nm, along with key image features including H_σ_ and H, play a vital role in fermentation degree discrimination. These features not only showed clear directional effects on model predictions but also reflected characteristic pigment transformation processes during tea fermentation. This discovery offers valuable insights for optimizing model performance and deepening our understanding of the underlying fermentation mechanisms.

By integrating image and spectral information across the entire fermentation process and interpreting model decisions through SHAP, this study achieves high-precision discrimination of fermentation stages. This not only provides a basis for developing non-destructive fermentation detection systems but also supplies a reliable technical route for digital transformation and intelligent advancement in tea processing. Although significant results have been achieved in this study, the model is based solely on sample data from specific seasons, varieties, and origins, and its generalization capability for direct application under other conditions requires further validation. Moreover, LSTM and GRU models employed fixed hyperparameters without optimization, resulting in their performance being inferior to that of the SVM model. To overcome these limitations and promote practical implementation, future work will focus on: (1) constructing an open benchmark dataset covering multiple production regions, seasons, and varieties to enhance model generalizability; (2) optimizing model hyperparameters and investigating algorithms such as domain adaptation and incremental learning to develop adaptive models capable of accommodating dynamic changes in environment and raw materials; (3) advancing the integration of algorithms with dedicated hardware and conducting long-term online validation and iteration on actual production lines, ultimately achieving industrial deployment of the technology.

## Figures and Tables

**Table 1:** Performance of models based on image features.

Data	Feature Selection or Dimensionality Reduction Methods	No. of Variables	Modeling Methods and Model Performance				
			Model	Train Acc	Test Acc	Test Accuracy 95% CI	Optimal Configuration (C, γ) for SVM; Epochs for LSTM/GRU
Image	None	26	SVM	89.29	76.39	66.67–84.72	(512, 0.001)
			LSTM	92.26	79.17	69.44–87.50	225
			GRU	90.48	77.78	68.06–87.50	250
	Pearson	13	SVM	90.48	80.56	70.83–88.89	(256, 0.004)
			LSTM	86.90	80.56	70.83–90.28	225
			GRU	88.10	80.56	71.53–90.28	250
	PCA	9	SVM	94.05	77.78	68.06–87.50	(512, 0.004)
			LSTM	94.64	80.56	70.83–88.89	245
			GRU	94.05	76.39	66.67–84.72	250

**Table 2:** Performance of models based on hyperspectral features.

Data	Feature Selection or Dimensionality Reduction Methods	No. of Variables	Data	Modeling Methods and Model Performance				
				Model	Train Acc	Test Acc	Test Accuracy 95% CI	Optimal Configuration (C, γ) for SVM; Epochs for LSTM/GRU
Spectra	Raw	CARS	18	SVM	89.29	80.56	72.22–88.89	(512, 0.002)
				LSTM	80.95	73.61	62.50–82.64	225
				GRU	82.14	75.00	65.28–84.72	200
	Nor		32	SVM	99.40	80.56	71.53–88.89	(128, 0.004)
				LSTM	93.45	81.94	73.61–90.28	245
				GRU	97.02	80.56	70.83–88.89	250
	SNV		28	SVM	99.40	81.94	72.22–90.28	(512, 0.002)
				LSTM	95.83	77.78	66.67–87.50	235
				GRU	97.62	80.56	70.83–88.89	250
	Raw	SPA	29	SVM	87.50	80.56	72.22–89.58	(512, 0.002)
				LSTM	79.17	70.83	61.11–80.56	240
				GRU	79.76	68.06	56.94–79.17	225
	Nor		24	SVM	99.40	87.50	79.17–94.44	(256, 0.004)
				LSTM	94.05	79.17	69.44–87.50	250
				GRU	91.07	79.17	69.44–87.50	185
	SNV		23	SVM	98.81	81.94	72.22–90.28	(256, 0.004)
				LSTM	93.45	81.94	72.22–90.28	245
				GRU	95.83	80.56	72.22–88.89	235
	Raw	PCA	9	SVM	93.45	79.17	69.44–87.50	(128, 0.004)
				LSTM	91.07	76.39	66.67–86.11	245
				GRU	88.10	75.00	65.28–84.72	240
	Nor		9	SVM	95.24	75.00	63.89–84.72	(256, 0.002)
				LSTM	92.26	79.17	69.44–88.89	245
				GRU	92.26	69.44	58.33–79.17	250
	SNV		6	SVM	89.29	79.17	69.44–87.50	(512, 0.002)
				LSTM	84.52	80.56	70.83–88.89	245
				GRU	86.90	79.17	69.44–88.89	245

**Table 3:** Performance of models based on fusion features.

Data	Feature Selection or Dimensionality Reduction Methods	No. of Variables	Data	Modeling Methods and Model Performance				
				Model	Train Acc	Test Acc	Test Accuracy 95% CI	Optimal Configuration (C, γ) for SVM; Epochs for LSTM/GRU
Fusion	Pearson+Nor-CARS	45(13+32)	14	SVM	95.24	93.06	87.50–97.92	(32, 0.004)
				LSTM	98.81	86.11	77.78–93.06	245
				GRU	97.62	86.11	77.78–93.06	250
	Pearson+SNV-CARS	41(13+28)	10	SVM	95.83	93.06	86.11–98.61	(16, 0.016)
				LSTM	97.62	90.28	83.33–97.22	250
				GRU	97.62	88.89	80.56–95.83	250
	Pearson+Nor-SPA	37(13+24)	15	SVM	98.81	94.44	88.89–98.61	(256, 0.004)
				LSTM	98.21	86.11	77.78–93.06	210
				GRU	98.81	84.72	76.39–93.06	250
	Pearson+SNV-SPA	36(13+23)	13	SVM	96.43	91.67	84.72–97.22	(16, 0.016)
				LSTM	98.81	84.72	76.39–93.06	250
				GRU	97.62	84.72	76.39–93.06	250
	PCA+Nor-PCA	18(9+9)	11	SVM	96.43	84.72	76.39–91.67	(128, 0.002)
				LSTM	97.62	80.56	72.22–88.89	245
				GRU	97.62	80.56	70.83–88.89	245
	PCA+SNV-PCA	15(9+6)	14	SVM	95.83	87.50	80.56–94.44	(16, 0.004)
				LSTM	100.00	84.72	76.39–93.06	245
				GRU	99.40	77.78	66.67–86.11	250

## Data Availability

The original contributions presented in this study are included in the article. Further inquiries can be directed to the corresponding author.
